# Selection and Optimization of Reference Genes for MicroRNA Expression Normalization by qRT-PCR in Chinese Cedar (*Cryptomeria fortunei*) under Multiple Stresses

**DOI:** 10.3390/ijms22147246

**Published:** 2021-07-06

**Authors:** Yingting Zhang, Jinyu Xue, Lijuan Zhu, Hailiang Hu, Junjie Yang, Jiebing Cui, Jin Xu

**Affiliations:** 1 Key Laboratory of Forest Genetics & Biotechnology of Ministry of Education, Nanjing Forestry University, Nanjing 210037, China; ytzhang@njfu.edu.cn (Y.Z.); xjinyu@njfu.edu.cn (J.X.); zhulijuanlucky@163.com (L.Z.); huhailiang@njfu.edu.cn (H.H.); yangjj@njfu.edu.cn (J.Y.); cuijiebing@163.com (J.C.); 2 Co-Innovation Center for Sustainable Forestry in Southern China, Nanjing Forestry University, Nanjing 210037, China; 3 College of Forestry, Nanjing Forestry University, Nanjing 210037, China

**Keywords:** miRNAs, reference gene, qRT-PCR, abiotic stress, hormone treatment, tissue

## Abstract

MicroRNA (miRNA) expression analysis is very important for investigating its functions. To date, no research on reference genes (RGs) for miRNAs in gymnosperms, including *Cryptomeria fortunei,* has been reported. Here, ten miRNAs (i.e., pab-miR159a, cln-miR162, cas-miR166d, pab-miR395b, ppt-miR894, cln-miR6725, novel1, novel6, novel14 and novel16) and three common RGs (*U6*, *5S* and *18S*) were selected as candidate RGs. qRT-PCR was used to analyse their expressions in *C. fortunei* under various experimental conditions, including multiple stresses (cold, heat, drought, salt, abscisic acid and gibberellin) and in various tissues (roots, stems, tender needles, cones and seeds). Four algorithms (delta Ct, geNorm, NormFinder and BestKeeper) were employed to assess the stability of candidate RG expression; the geometric mean and RefFinder program were used to comprehensively evaluate RG stability. According to the results, novel16, cln-miR6725, novel1 and *U6* were the most stable RGs for studying *C. fortunei* miRNA expression. In addition, the expression of three target miRNAs (aly-miR164c-5p, aly-miR168a-5p and smo-miR396) was examined to verify that the selected RGs are suitable for miRNA expression normalisation. This study may aid further investigations of miRNA expression/function in the response of *C. fortunei* to abiotic stress and provides an important basis for the standardisation of miRNA expression in other gymnosperm species.

## 1. Introduction

Chinese cedar (*Cryptomeria fortunei*) belongs to *Cupressaceae*. Because of its rapid growth, straight trunk and good wood texture, *C. fortunei* has become one of the main fast-growing timber afforestation species in subtropical high-elevation areas in China and has broad application prospects. However, *C. fortunei* usually grows in warm and humid climates, and its growth is often affected by adverse environmental conditions, such as low temperature, acid/aluminium and other stresses [1,2,3]. To date, molecular biology studies of *Cryptomeria* have largely focused on analyses of functional genes to reveal the growth and development mechanisms of these trees, whereas few studies have examined microRNAs (miRNAs) [4,5,6].

MiRNAs, with a length of approximately 20–24 nucleotides, are a type of endogenous single-stranded noncoding small RNA (sncRNA) involved in almost all biological processes, including growth and development processes, hormone signal transduction and various kinds of biotic and abiotic stress responses [7,8,9]. By regulating target genes, miRNAs play a vital role in the posttranscriptional or translational regulation of gene expression [8,10,11,12]. The study of gene expression patterns is the basis of molecular biology research, and it has become an important method to reveal gene levels and investigate growth-regulation mechanisms. Indeed, analysing miRNA expression patterns is crucial for identifying complex biological processes in plants, such as the mechanisms underlying adaptation to (a)biotic stresses and signal transduction pathways under stress.

Quantitative real-time polymerase chain reaction (qRT-PCR), currently one of the most commonly used methods for studying plant miRNA expression, has the characteristics of good reproducibility, strong specificity, excellent sensitivity, high efficiency and convenient operation [13,14,15]. Effective qRT-PCR data depend on many factors, such as the quality of the extracted RNA, the efficiency of the reverse transcription reaction, primer specificity and data processing methods [16,17]. To date, various strategies have been used to standardise qRT-PCR data, and using internal controls or RGs has become the most reliable method [18]. In general, the expression level of an ideal RG should not be affected by the species, tissue, experimental conditions or other factors; that is, expression levels under all environmental conditions should be relatively constant [19]. Unfortunately, there is no absolutely stably expressed gene, and the so-called stable expression of any internal RG is only stable in specific tissues or under specific environmental conditions. Therefore, it is necessary to strictly select suitable internal RGs for miRNAs when performing miRNA qRT-PCR experiments.

The internal RGs commonly used for miRNAs are mostly genes with that are short fragments (length < 2000 bp), such as *a small nuclear RNA* (*U6*), *18S* ribosomal RNA (rRNA) (*18S*), *5S* rRNA (*5S*), *5.8S* rRNA (*5.8S*) and *glyceraldehyde 3-phosphate dehydrogenase* (*GAPDH*) [20,21]. Nevertheless, according to an increasing number of experiments, these genes still have certain defects with regard to the applicability and accuracy of miRNA expression. More specifically, RGs selected from among conserved or novel miRNAs may be more stable than *U6*, *5S*, *5.8S* and protein-coding genes [22]. Although suitable internal RGs for miRNAs have been reported for only a few angiosperm plants, such as sugarcane (*Saccharum* spp.) [21], cucumber (*Cucumis sativus*) [23], wheat (*Triticum aestivum*) [24], longan tree (*Dimocarpus longan*) [25] and peach (*Prunus persica*) [26], there is still no report on the systematic evaluation of RGs for studying miRNAs in gymnosperms, including *C. fortunei,* to date.

In this study, we selected thirteen candidate RGs, including three commonly used RGs (*U6*, *5S* and *18S*) and ten stable and highly expressed miRNAs (pab-miR159a, cln-miR162, cas-miR166d, pab-miR395b, ppt-miR894, cln-miR6725, novel1, novel6, novel14 and novel16), and used qRT-PCR to systematically analyse their expression stability under multiple treatments (including cold-, heat-, drought-, salt-, abscisic acid (ABA)- and gibberellin (GA_3_)-treated samples) and in different tissues (roots, stems, tender needles, cones and seeds). Four algorithms (delta Ct, geNorm, NormFinder and BestKeeper) were utilised for data analysis, and the geometric mean and ReFinder network program were applied to comprehensively determine the most stable gene among these RGs for *C. fortunei*. In addition, the expression of three target miRNAs, i.e., aly-miR164c-5p, aly-miR168a-5p and smo-miR396, was used to verify that the selected RGs are suitable for gene-expression normalisation in different tissues or under the selected treatment. These results identified appropriate RGs that can be used to normalise the expression of miRNAs in *C. fortunei*, providing a basis for normalising miRNA expression in other coniferous (gymnosperm) species.

## 2. Results

### 2.1. Assessment of Primer Specificity and PCR Amplification Efficiency

**Figure 1:**
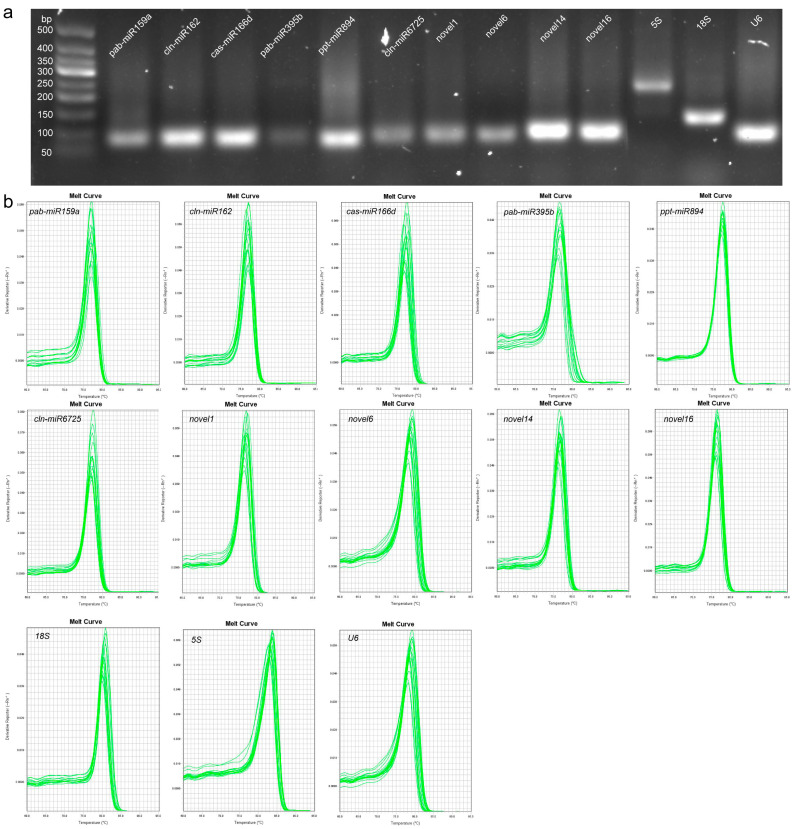
Specificity of each candidate reference gene (RG) primer pair. (**a**) Confirmation of the specificity of qPCR primer amplification of candidate RGs by agarose gel electrophoresis. (**b**) Melting-curve analysis of quantitative real-time PCR (qRT-PCR) amplification of 13 candidate RGs in *Cryptomeria fortunei*.

A total of thirteen candidate RGs, including ten miRNAs and three RNAs, were selected for gene-normalisation studies in different samples by qRT-PCR analysis. Melting curves and 2.5% (*w*/*v*) agarose gel electrophoresis showed that each pair of primers used to amplify a candidate RG produced a single PCR-specific product of the desired size (Figure 1), indicating that all primer pairs used for RG selection had good primer specificity for PCR amplification. The amplification efficiency (E) values of all candidate genes ranged from 90.04% (novel6) to 114.97% (pab-miR159a), and regression analysis of all primer pairs showed an correlation coefficient (R^2^) ≥ 0.978 (Table 1), indicating a strong correlation between the Ct values detected in all amplification reactions and the relative amount of template. Therefore, these candidate RGs were used for further analysis.

### 2.2. Expression Levels of Candidate RGs

**Figure 2:**
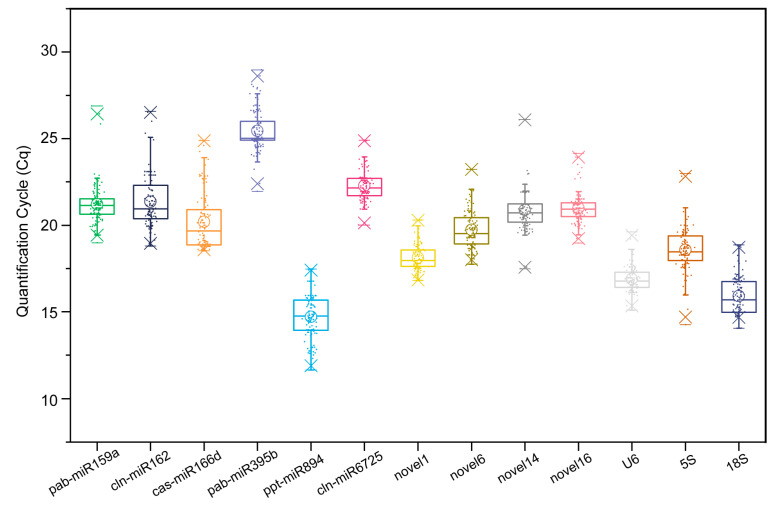
Quantification cycle values of 13 candidate reference genes in different tissues and under multiple stresses. The box indicates the 25th and 75th percentiles, with the line across the box representing the median. Whiskers and asterisks represent 99% confidence intervals and outliers, respectively. Upper and lower horizontal lines indicate the maximum and minimum values, respectively, and small circles represent the average values.

To investigate the applicability of these thirteen candidate RGs, we analysed the expression levels of candidate RGs under multiple stresses (heat, cold, drought, salt, GA_3_ and ABA) and in different tissues (stems, roots, seeds, cones and tender needles). These candidate RGs exhibited a wide expression range, with Ct values ranging from 14.716 (ppt-miR894) to 25.437 (pab-miR395b) (Figure 2). Compared to the other candidate RGs, ppt-miR894 (14.716), *18S* (15.910) and *U6* (16.888) had relatively high expression levels; conversely, pab-miR159a (21.209), cln-miR162 (21.357), cln-miR6725 (22.267) and pab-miR395b (25.437) were expressed at relatively low levels (Figure 2). Moreover, novel1 expression was the least variable (3.500 Ct; the maximum and minimum Ct values were 20.306 and 16.806, respectively) among all 123 samples, followed by that of *U6* (4.515). Furthermore, *5S* expression showed the greatest variability (8.723 Ct), ranging from 14.250 to 22.974 (Figure 2). Therefore, novel1 and *U6* can initially be considered two stable RGs, though further analysis is required.

### 2.3. Expression Stability of Candidate RGs under Multiple Stresses and in Different Tissues

To identify the most suitable RGs for gene-expression analysis under cold, heat, drought and high-salinity stresses, ABA and GA_3_ treatments and in different tissues of *C. fortunei*, the expression stability of candidate RGs was evaluated using four different algorithms.

#### 2.3.1. Delta Ct Method

**Figure 3:**
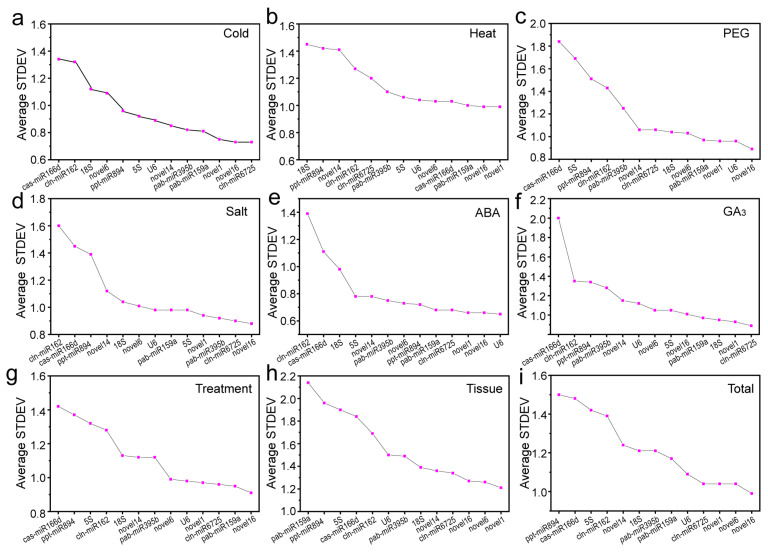
Average standard deviation (STDEV) by delta Ct analysis. Results from (**a**) 4 °C cold stress; (**b**) 40 °C heat stress; (**c**) drought stress simulated by 15% PEG-6000 treatment; (**d**) salt stress stimulated by 200 mM NaCl treatment; (**e**) 200 μM ABA treatment; (**f**) 200 μM GA_3_ treatment; (**g**) multiple stresses; (**h**) different tissues (roots, stems, tender needles, cones and seeds); (**i**) total samples.

A simple delta Ct method was employed to compare the relative expression of “gene pairs” in each group of samples and rank the stability of candidate RGs based on the reproducibility of the average standard deviation (STDEV) of gene-expression differences between samples [27], wherein a smaller STDEV indicated more stable gene expression. For GA_3_- or cold-treated samples, cln-miR6725 was more stable than other RGs; in samples treated with ABA, *U6* showed the strongest stability (Figure 3). Under high-temperature conditions or in different tissues, novel1 had the most stable expression levels. However, novel16 showed the lowest delta Ct values for drought- or salt-treated samples, as well as under multiple stresses and in all samples, with greater expression stability than other genes (Figure 3). Notably, cas-miR166d and cln-miR162 were the least stable under most conditions (Figure 3).

#### 2.3.2. GeNorm Analysis

**Figure 4:**
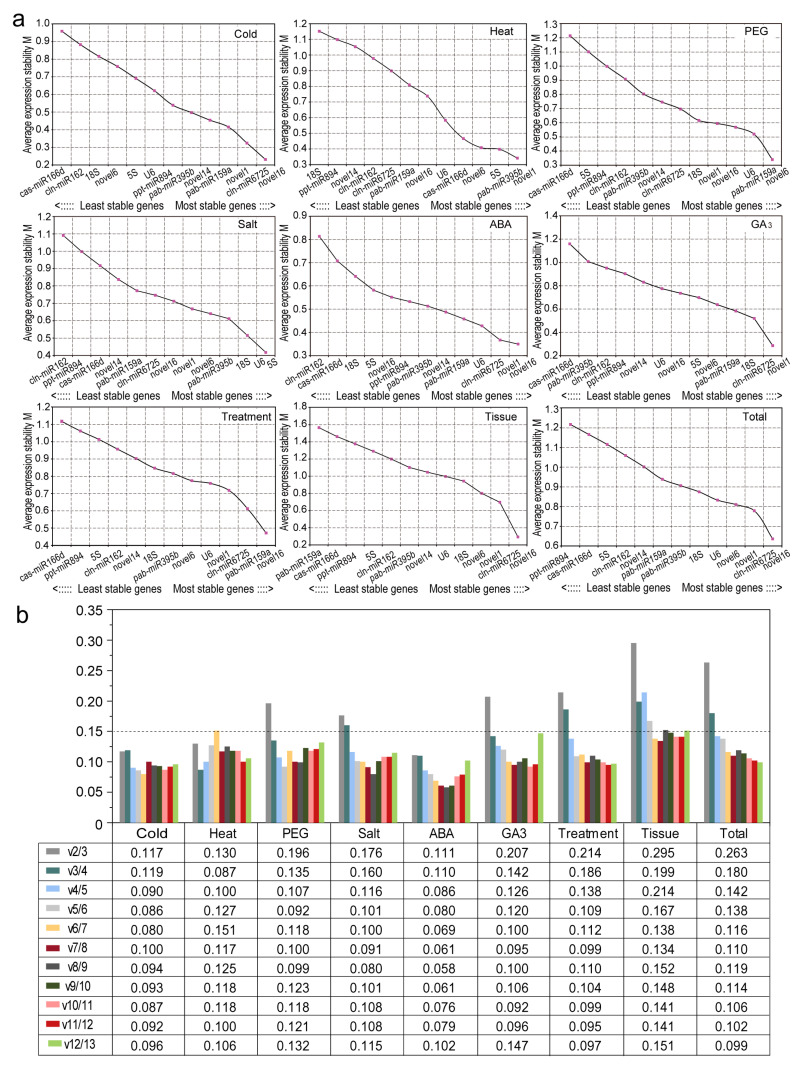
Average expression stability values and pairwise variations by geNorm analysis. (**a**) Expression-stability values (M) and rankings of the 13 candidate reference genes of *C. fortunei* calculated using geNorm. The most and least stable genes are on the right and left, respectively. (**b**) Determination of the optimal number of reference genes for *C. fortunei*.

The geNorm program calculates the expression stability (M) value of the stability of each candidate RG [28], which can be used to screen any tested internal RG (combination) to correct data and make relative quantitative results more accurate. The M value is set to 1.5 as the cut-off, and in general, the gene with the lowest M value has the highest stability, whereas the gene with the highest M value has the least stable expression. As shown in Figure 4a, the M values of novel16 and cln-miR6725 were lower than those of the other genes under cold stress, suggesting that they are the most stable candidate RGs, similar to the results found for the sample sets of different tissues and all samples. In addition, novel1 and pab-miR395b were more stable than other RGs under heat stress; pab-miR159a and novel6 as well as *5S* and *U6* were the two most stably expressed RGs in drought and salt treatments, respectively. In samples treated with ABA, novel16 and novel1 had greater expression stability than other genes; novel1 and cln-miR6725 exhibited stable expression in GA_3_-treated samples. Under multiple stresses, novel16 and pab-miR159a showed the strongest stability. In contrast, ppt-miR894 and cas-miR166d were the least stable in most cases (Figure 4a).

Although most studies only use a single gene as a standardised internal control, multiple RGs may produce more reliable results. Therefore, we calculated pairwise changes (V_n_/V_n+1_) using geNorm with a threshold of 0.15 to determine the optimal number of RGs for each group of samples [28]. For cold-, heat- or ABA-treated samples, V_2_/_3_ values were all < 0.15 (0.117, 0.130 and 0.111, respectively), indicating that two RGs were sufficient for normalisation in each group. Under polyethylene glycol (PEG)-simulated drought stress or GA_3_ treatment, V_3_/_4_ values were less than 0.15 (0.135 and 0.142, respectively); thus, at least three genes were needed for normalisation. Under salt stress or multiple stresses, as well as in all samples, V_4_/_5_ values were < 0.15, with four RGs being required to obtain accurate results. In different tissues, the V_6_/_7_ value was lower than 0.15 (0.138), and six RGs were optimal for standardisation (Figure 4b).

#### 2.3.3. Norm Finder Analysis

To confirm the results obtained using geNorm software, we calculated stability values through NormFinder to evaluate the stability of the expression of these 13 candidate RGs, with low stability values indicating high expression stability [29]. Notably, under cold-, heat-, drought-, salt- or multiple stresses, as well as in all total samples, novel16 was the most stable gene. The best combination of RGs for the six groups were novel16 + novel1, novel16 + pab-miR159a, novel16 + *U6* + novel1, novel16 + cln-miR6725 + pab-miR395b + novel1, novel16 + pab-miR159a + cln-miR6725 + novel1 and novel16 + cln-miR6725 + novel1 + novel6. Moreover, cln-miR6725, *U6* and novel1 had the strongest stability across GA_3_-treated, ABA-treated and different tissue samples, respectively (Table 2). However, in most cases, cas-miR166d and cln-miR162 were the least stable (Table 2).

#### 2.3.4. BestKeeper Algorithm

**Figure 5:**
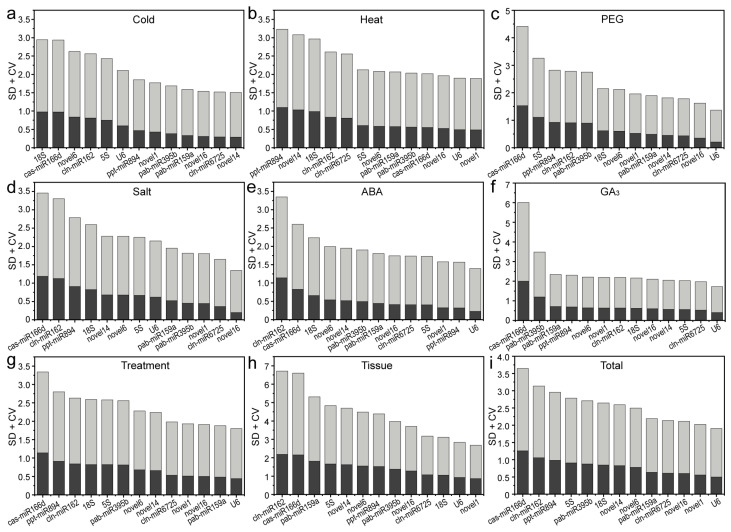
Stacked histograms showing the rankings of 13 candidate reference genes using the BestKeeper algorithm. Results from (**a**) 4 °C cold stress; (**b**) 40 °C heat stress; (**c**) drought stress simulated by 15% PEG-6000 treatment; (**d**) salt stress stimulated by 200 mM NaCl treatment; (**e**) 200 μM ABA treatment; (**f**) 200 μM GA_3_ treatment; (**g**) multiple stresses; (**h**) different tissues (roots, stems, tender needles, cones and seeds); (**i**) total samples. Dark grey bars represent standard deviations (SDs) of average Ct values, and light grey bars represent coefficients of variance (CVs).

BestKeeper ranks candidate RGs based on the coefficient of variation (CV) and standard deviation (SD) of the average Ct value. The most stable genes showed the lowest SD ± CV values; the SD value was also < 1 [16]. For drought-, ABA- or GA_3_-treated samples, *U6* showed the highest stability under multiple stresses and in all tested experimental conditions; novel1, novel14 and novel16 had the most stable expression levels under heat stress or in different tissues and under cold stress and salt stress (Figure 5). Similar to the NormFinder results, cas-miR166d and cln-miR162 were the least stable RGs in most cases (Figure 5).

### 2.4. Comprehensive Stability Analysis of RGs

**Figure 6:**
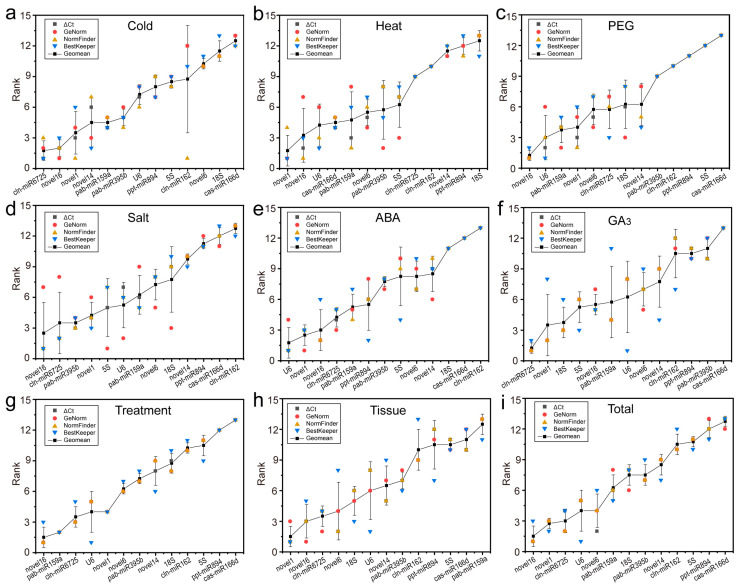
Comprehensive ranking of 13 RGs calculated as the geometric means of four types of rankings. Results from (**a**) 4 °C cold stress; (**b**) 40 °C heat stress; (**c**) drought stress simulated by 15% PEG-6000 treatment; (**d**) salt stress stimulated by 200 mM NaCl treatment; (**e**) 200 μM ABA treatment; (**f**) 200 μM GA_3_ treatment; (**g**) multiple stresses; (**h**) different tissues (roots, stems, tender needles, cones and seeds); (**i**) total samples.

We performed comprehensive ranking analysis based on the results derived from the geometric mean of these four algorithms and selected the optimal number of RGs according to the results of GeNorm, thereby determining the best RGs (combination), as shown in Figure 6. Under cold stress, the best internal reference combination was cln-miR6725 + novel16. For 40 °C-treated samples, the optimal reference pair was novel1 + novel16. Under PEG-simulated drought stress, novel16 + *U6* + pab-miR159a was the best internal reference combination. For salt-treated samples, novel16 + cln-miR6725 + pab-miR395b + novel1 had the most stable performance. In samples treated with ABA or GA_3_, *U6* + novel1 and cln-miR6725 + novel1 + *18S* showed the strongest stability, respectively. Under multiple stresses, novel16 + pab-miR159a + *U6* + cln-miR6725 was the most stable. In various tissues, novel1 + novel16 + cln-miR6725 + novel6 + *18S* + *U6* had the most stable expression levels. Furthermore, novel16 + cln-miR6725 + novel1 + *U6* showed the strongest stability in all samples.

**Figure 7:**
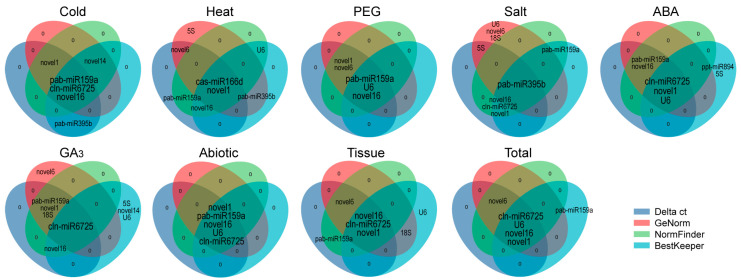
The top 5 most stable reference genes (RGs) were generated by delta-Ct, geNorm, NormFinder and BestKeeper. Blue, pink, green and sky-blue circles each contain the top 5 most stable RGs identified with delta-Ct, geNorm, NormFinder and BestKeeper, respectively. Genes in the overlapping area were confirmed as the top 5 most stable RGs by more than one algorithm.

The RefFinder program was also utilised to verify the comprehensive ranking results of the candidate RGs, which were basically the same as those obtained using the geometric mean, with only slight differences for salt- or ABA-treated samples (Table 3). For example, novel16 + cln-miR6725 + pab-miR395b + *5S* was the best RG combination under salt stress, with only *5S* showing a difference; *U6* + novel16 was the best RG combination for ABA-treated samples, with only novel16 showing a difference. We found these stable RGs selected under various experimental conditions to basically be the top 5 most stable genes selected using the four software programs (at least three software programs) (Figure 7). In addition, statistical analysis of the two RGs with the lowest stability rankings showed that cas-miR166d was the least stable RG (Figure 6).

### 2.5. Validation of the Stability of RGs

**Figure 8:**
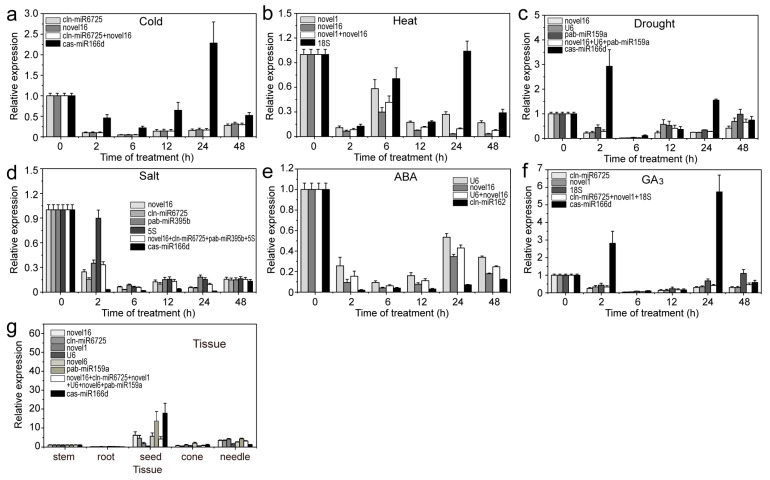
Relative expression levels of aly-miR164c-5p under different experimental conditions. Results for (**a**) 4 °C cold stress; (**b**) 40 °C heat stress; (**c**) drought stress simulated by 15% PEG-6000 treatment; (**d**) salt stress stimulated by 200 mM NaCl treatment; (**e**) 200 μM ABA treatment; (**f**) 200 μM GA_3_ treatment; (**g**) different tissues (roots, stems, tender needles, cones and seeds). Error bars represent standard deviations (SD) (n = 3).

**Figure 9:**
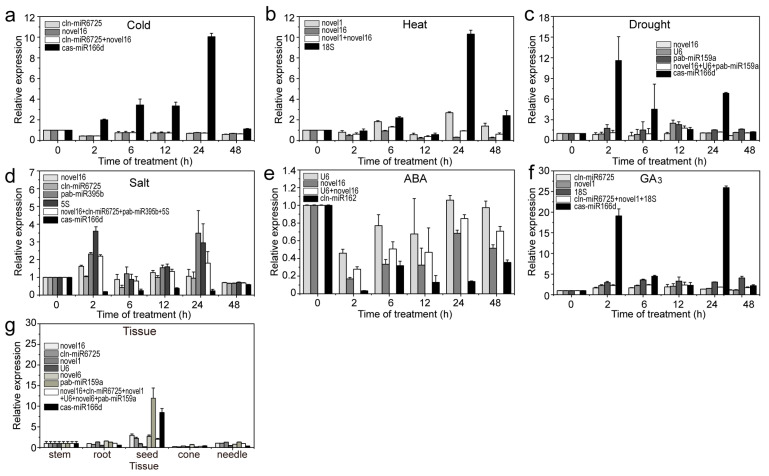
Relative expression levels of aly-miR168a-5p under different experimental conditions. Results from (**a**) 4 °C cold stress; (**b**) 40 °C heat stress; (**c**) drought stress simulated by 15% PEG-6000 treatment; (**d**) salt stress stimulated by 200 mM NaCl treatment; (**e**) 200 μM ABA treatment; (**f**) 200 μM GA_3_ treatment; (**g**) different tissues (roots, stems, tender needles, cones and seeds). Error bars represent standard deviations (SD) (n = 3).

**Figure 10:**
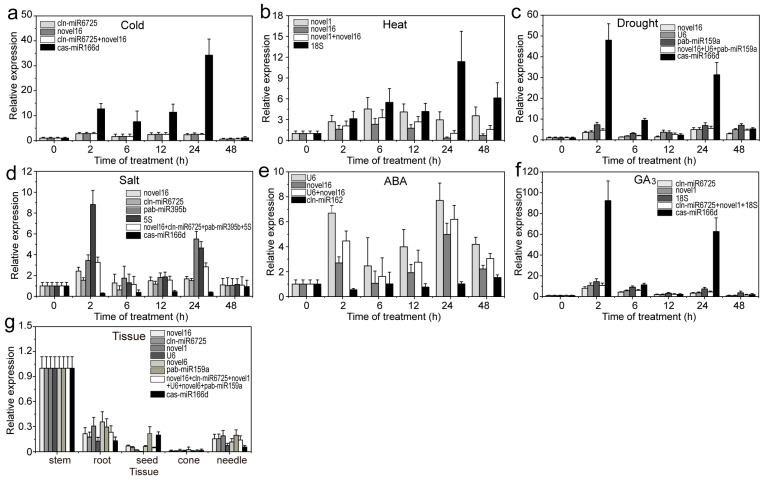
Relative expression levels of smo-miR396 under different experimental conditions. Results from (**a**) 4 °C cold stress; (**b**) 40 °C heat stress; (**c**) drought stress simulated by 15% PEG-6000 treatment; (**d**) salt stress stimulated by 200 mM NaCl treatment; (**e**) 200 μM ABA treatment; (**f**) 200 μM GA_3_ treatment; (**g**) different tissues (roots, stems, tender needles, cones and seeds). Error bars represent standard deviations (SD) (n = 3).

To verify the stable RG expression accuracy, three miRNAs (aly-miR164c-5p, aly-miR168a-5p and smo-miR396) with relatively high abundance (transcripts per million (TPM) value > 10) were used as targets for qRT-PCR analysis. When using stable or unstable RGs, aly-miR164c-5p, aly-miR168a-5p and smo-miR396 were all found to be highly expressed in stems or seeds; however, expression levels were often misestimated when using unstable RGs compared to stable RGs (Figure 8, FIgure 9, Figure 10). For example, when using unstable RGs, aly-miR164c-5p expression was 2.894-, 3.776-, 9.582-, 42.589-, 3.130-, 1.304- and 4.187-fold that when using stable internal RGs (novel1, novel16, cln-miR6725, novel6, *18S* or *U6*) or a combination (novel1 + novel16 + cln-miR6725 + novel6 + *18S* + *U6*), respectively (Figure 8).

Additionally, the expression of aly-miR164c-5p was found to be downregulated under different treatments when using the stable RGs compared to the control (untreated samples). In contrast, aly-miR164c-5p was significantly upregulated in the cold (24 h), heat (24 h), drought (2 h and 24 h) and GA_3_ (2 h and 24 h) treatments when using unstable RGs; under other treatments (ABA and salt), the expression levels of aly-miR164c-5p were underestimated using unstable RGs compared to those obtained with stable RGs (combination) (Figure 8). Under cold treatment (2–48 h), the expression of aly-miR168a-5p was downregulated when using stable RGs compared to control levels, though expression of aly-miR168a-5p was upregulated when using unstable internal control genes (Figure 9). Similarly, under cold treatment (24 h), the expression of smo-miR396 was slightly upregulated compared to the control when using stable RGs, whereas that of smo-miR396 was significantly upregulated when using unstable RGs (Figure 10). Under salt (2–24 h) and ABA (2 h, 12–48 h) treatments, the expression levels of smo-miR396 and aly-miR168a-5p were downregulated when using unstable RGs; in addition, under heat/drought/GA_3_ treatments, the expression levels of smo-miR396 and aly-miR168a-5p were often underestimated when using unstable RGs (Figure 9 and Figure 10). In general, using different RGs to correct the expression level of target genes will lead to different results, i.e., if RGs are incorrectly selected, the relative expression of a target gene may be incorrectly estimated.

## 3. Discussion

MiRNA expression analysis is very important for studying miRNA function in biological research. Many methods have proven useful for studying the expression levels of miRNAs, and qRT-PCR has become a widely used method to examine gene-expression patterns. However, accurate qRT-PCR analysis of miRNAs is challenging due to the limited flexibility of primer design and the lack of appropriate RGs to standardise miRNAs [30]. The most commonly used RGs in plant miRNA qRT-PCR are still *U6* and *5S* [20,21]. Previous studies have shown that certain miRNAs have higher stability than mRNAs, as in rice (*Oryza sativa*) [30], sweet potato (*Ipomoea batatas*) [31] and peach [26]. Unfortunately, there are no universal RGs [19]. If a common internal RG is selected without screening, it is likely to reduce the accuracy of quantitative analysis results and even result in incorrect conclusions. Therefore, it is necessary to strictly screen genes before their use as RGs to ensure the accuracy of qRT-PCR analysis. Nevertheless, research on reference miRNAs in *C. fortunei* has not yet been reported. To improve the accuracy of gene-expression research, we systematically selected reliable internal control genes for the normalisation of miRNA expression in multiple tissues or under multiple stresses in *C. fortunei*.

In this study, the E values of the thirteen candidate RG primer pairs ranged from 90.04% to 114.97%, and R^2^ values were 0.978−0.999 (Table 1). These results indicate that the primer pairs used for RG selection have high accuracy, efficiency and sensitivity. Additionally, the average Ct values of the candidate RGs ranged from 14.716 (ppt-miR894) to 25.437 (pab-miR395b) (Figure 2). This is similar to the results for sweet potato [31] and wheat [24], in which these candidate RGs showed different expression levels with various test materials, with average Ct values from 6.7 to 33.5. These results indicate that in a given sample, no RG has constant expression levels across different conditions; accordingly, it is very important to select suitable RGs for miRNA standardisation under specific experimental conditions.

We used four common algorithms (delta Ct, geNorm, NormFinder and BestKeeper) to evaluate and determine stable RGs and found that the top five genes selected by various algorithms were generally similar (Figure 7; Table 3). For example, for all samples, novel16, novel6, novel1, cln-miR6725 and *U6* were the top five genes based on delta Ct, geNorm and NormFinder analyses; BestKeeper analysis showed similar results, except for novel6. Regardless, the internal rankings of stability genes produced by different algorithms under each experimental condition were quite different (Figure 3, Figure 4 and Figure 5; Table 2). For example, among all samples, novel16 and novel6 ranked first and second in delta Ct, first and fourth in geNorm (NormFinder), and third and sixth in BestKeeper (Table 2). Such a result was also observed in the selection of miRNAs for other plants, such as wheat [24] and peach [26], for which stability level differences were generated by the algorithms due to their different calculation approaches applied and their sensitivity to coregulated candidates.

Therefore, in actual applications, it is necessary to comprehensively consider the results provided by these algorithms. We used the geometric mean of the rankings combined with RefFinder to comprehensively analyse the stability of RGs and finally obtained the best RG (combination), as based on the best RG number provided by geNorm analysis. Fortunately, we found that the results of the geometric mean method in each treatment and different tissues were similar to those in RefFinder, showing that the results of software analysis alone were accurate (Figure 6; Table 3). The results also indicated that the following genes could be used as RGs under various experimental conditions: for cold stress, the best internal reference combination was cln-miR6725 + novel16; for 40 °C-treated samples, the optimal reference pair was novel1 + novel16; for PEG-simulated drought stress, novel16 + *U6* + pab-miR159a was the best internal reference combination; for salt-treated samples, novel16 + cln-miR6725 + pab-miR395b + 5S had the most stable performance; for samples treated with ABA or GA_3_, *U6* + novel16 and cln-miR6725 + novel1 + *18S* showed the strongest stability (Table 3). Under multiple stresses, novel16 + pab-miR159a + *U6* + cln-miR6725 was the most stable. In various tissues, novel1 + novel16 + cln-miR6725 + novel6 + *18S* + *U6* had the most stable expression levels; in all samples, novel16 + cln-miR6725 + novel1 + *U6* displayed the strongest stability (Table 3). Furthermore, certain differences in the optimal combination of RGs in different treatments/tissues of the same species exist. Research on such differences in RGs has also been reported for poplar (*Populus*) [20], wheat [24], tea plant (*Camellia sinensis*) [32] and other plants.

Although there was no RG that was always the best choice under the various experimental conditions, novel16, cln-miR6725, novel1 and *U6* are proposed as good candidates for *C. fortunei* miRNA expression studies (Figure 6; Table 3). We divided all samples into different subsets (treatment and tissue samples) and found that the optimal genes under each experimental environment included at least two or more of these four genes (novel16, cln-miR6725, novel1 and *U6*) (Figure 6; Table 3), confirming the applicability of these genes in *C. fortunei*. Two novel miRNAs (novel16 and novel1) and cln-miR6725 have rarely been reported as internal control genes in plants, though *U6* is a common RG widely used for miRNA standardisation in qRT-PCR and is reported as the most stable gene in RG selection studies of Valencia sweet orange (*Citrus sinensis*) [33] and tea plant [32]. *U6* is not found to be a suitable internal reference during longan somatic embryogenesis [25]. In contrast, cas-miR166d showed the most unstable expression in *C. fortunei* under most experimental conditions. This RG also has unstable expression patterns in cold-treated tomato (*Solanum lycopersicum*) seedlings [34] and winter turnip rape (*Brassica rapa*) leaves and roots [35], while miR166a is the most appropriate for normalisation in tamarillo (*Solanum betaceum*) callus samples [36]. These results can be clarified by the fact that RGs exhibit specific expression patterns, with large differences in applicability.

MiR164, MiR168 and MiR396 can regulate the expression levels of plant genes and ultimately affect plant growth and development and respond to (a)biotic stresses [36,37,38,39,40]. A large number of studies have shown significant differences in the expression of target genes after normalisation with stable and unstable RGs [9,41,42]. In the present study, we used stable and unstable RGs to investigate the expression patterns of target miRNAs (aly-miR164c-5p, aly-miR168a-5p and smo-miR396) under various stresses or in different tissues; inappropriate candidate RGs were assessed, which resulted in the incorrect estimation of the expression (underestimation/overestimation) or expression trends of miRNAs. This confirms the importance of selecting appropriate RGs in molecular biology analyses.

## 4. Materials and Methods

### 4.1. Plant Materials and Treatments

In June 2014, a well-growing *C. fortunei* tree located in Xikou Forest Farm (118°32′ E, 25°23′ N), Xianyou, Fujian Province, China, was chosen as the mother tree, and semilignified branches with 2–3 lateral buds were taken to establish cuttings (12−16 cm); the cuttings were cut straight at the upper incision and at a 45° oblique angle at the lower incision. The cuttings were soaked in distilled water for 12 h, and then soaked with 0.1 g L^−1^ GGR 6 rooting powder (Beijing Aibiti Biological Technology Co., Ltd., Beijing, China) for 4 h to promote rooting. The treated cuttings were cut in black plastic pots (30 cm and 25 cm in diameter and height, respectively) with mixed soil substrate (vermiculite:perlite:yellow sand, 1:2:2, *v*:*v*:*v*) and placed in the greenhouse of the Baima Teaching and Research Base of Nanjing Forestry University (31°37′ N, 119°11′ E), Nanjing, Jiangsu Province, China.

In October 2020, eighteen six-year-old *C. fortunei* #3 cutting seedlings (CSs) displaying robust and consistent growth were selected and divided into six groups. Multiple stress treatments were applied to the CSs as follows [15,43]. For treatments of cold or heat, the CSs were transferred to an incubator (MLR 351H, SANYO Electric Co., Ltd., Osaka, Japan) set to 4 °C or 40 °C. Drought and salt stresses were simulated by the application of 15% (*w*/*v*) PEG-6000 and 200 mM sodium chloride (NaCl) in 1/4 Hoagland solution (1 L per plant), respectively. For hormone treatments, the CSs were sprayed with 200 μM ABA or 200 μM GA_3_ evenly until all the needles were completely wet. Except for the CSs under low- and high-temperature stresses, the plants were cultivated in an incubator at 25 °C; all CSs were cultivated in a light incubator with the same photoperiod (12 h light/12 h dark cycle) and relative humidity (60%). Except for heat-treated samples (tender needles) taken at 0.5, 1, 2, 4 and 6 h during prolonged 40 °C treatment, tender needle samples were taken at 0, 2, 6, 12, 24 and 48 h during each stress treatment. Different tissue samples (roots, stems, tender needles, cones and seeds) of *C. fortunei* were collected from tree #3, which had been growing in the natural environment for 15 years. Three independent biological replicates were performed for each sample. All collected samples were quickly frozen in liquid nitrogen and stored at –80 °C until miRNA extraction.

### 4.2. Selection of Candidate RGs and Primer Design

A pair of *U6* primers was obtained from Kou et al. [33], and two pairs of gene primers (*5S* and *18S*) were designed using Primer Premier 5.0 software (Premier Biosoft International, Palo Alto, CA, USA). The parameters were as follows: PCR product length of 70–250 bp, dissolution temperature of 58–62 °C and CG content of 40–60%. We used the Primer-BLAST tool (http://www.ncbi.nlm.nih.gov/tools/primer-blast/ (accessed on 18 June 2012)) at NCBI for the specific detection of plant primers. *C. fortunei* needle miRNAs were sequenced by high-throughput technology (BioProject under accession PRJNA720228), and ten miRNAs with abundant and stable expression levels (TPM value > 30 and a fold change in expression < 2) were selected as candidates (Table S1). Forward primers for these miRNAs were based on the mature miRNA sequence, with U replaced with T. Bases were then added or subtracted at both ends of the primers to achieve the appropriate Tm value at 65 °C and a GC content of 40–60% (https://pubmed.ncbi.nlm.nih.gov/10868275/ (accessed on June 2000)) (Table 1); miRNA reverse primers were obtained with a miRcute miRNA qPCR Detection Kit (SYBR Green) (Tiangen Biotech Co., Ltd., Beijing, China). All primers were synthesised by Tsingke Biotech Co., Ltd. (Nanjing, Jiangsu, China).

### 4.3. RNA Extraction and cDNA Preparation

MiRNAs were extracted from 0.1 g samples using a miRNA Isolation Kit (Tiangen Biotech Co., Ltd., Beijing, China) according to the manufacturer’s instructions. MiRNA integrity, purity and concentration were assessed using 2.5% (*w*/*v*) agarose gel electrophoresis and spectrophotometry (NanoDrop 2000, Thermo Scientific, Wilmington, DE, USA), respectively. For qualified samples, first-strand cDNAs were synthesised from 1 µg miRNA, according to the procedures of a miRcute Plus miRNA First-Strand cDNA Kit (Tiangen Biotech Co.) using the Poly(A) method. Specifically, a 20-µL reaction system (1 µg miRNAs, 10 µL 2 × miRNA RT Reaction Buffer and 2 µL miRNA RT Enzyme Mix were blended to a total volume of 20 µL with RNase-free water) was subjected to 42 °C for 60 min and 95 °C for 3 min. All synthesised cDNAs were immediately stored at −20 °C until used.

### 4.4. RT-PCR and qRT-PCR Analysis

To verify the accuracy of the designed primers, RT-PCR amplification was performed using a Fast PCR Kit (Vazyme Biotechnology Co., Ltd., Nanjing, Jiangsu, China). The 20 µL reaction system was as follows: 2 µL of each primer pair (10 μM forward and reverse primers), 1 µL cDNA, 10 µL 2 × Rapid Taq Master Mix and 7 µL ddH_2_O. The reaction settings were as follows: 95 °C for 3 min, followed by 35 cycles of 95 °C for 15 s, 60 °C for 15 s and 72 °C for 15 s, and a final extension at 72 °C for 5 min. The PCR products were detected by 2.5% (*w*/*v*) agarose gel electrophoresis.

To monitor the E value, 4 µL of the cDNA template from all samples was mixed uniformly and serially diluted 5-fold (1:4, 1:24, 1:124, 1:624, 1:3124, cDNA:water, *v*:*v*). At each dilution, qRT-PCR was performed for each pair of primers to obtain Ct values and to establish a standard curve; the R^2^, slope and E values were calculated using Microsoft Office Excel 2007 (Microsoft Co., Redmond, WA, USA). The following modified equation was applied: E = (5^−1/slope^ − 1) × 100%.

All qRT-PCRs were performed with an Applied Biosystems (ABI) 7500 real-time PCR system (ABI, Foster City, CA, USA), with the following amplification procedure: 95 °C predenaturation for 15 min, followed by 40 cycles of 94 °C for 20 s (denaturation) and 60 °C for 34 s (annealing extension); a melting curve was generated at 60−95 °C. The 20-µL qRT-PCR system, i.e., 10 µL 2 × miRcute Plus miRNA Premix, 0.8 µL of each primer pair (10 μM forward and reverse primers), 2 µL 10-fold diluted cDNA (cDNA:water, 1:9, *v*:*v*), 1.6 µL 50 × ROX Reference Dye and 5.6 µL RNase-Free ddH_2_O, was prepared according to the instructions of a miRcute Plus miRNA qPCR Detection Kit (SYBR Green) (Tiangen Biotech Co.). A negative control without the addition of each candidate internal RG was used to test for background amplification. Three technical replicates were performed for each sample, and the mean was used for qRT-PCR analysis.

### 4.5. Gene Expression Stability Analysis

Four different algorithms (i.e., delta Ct [27], geNorm (version 3.5) [28], NormFinder (version 0.953) [29] and BestKeeper (version 1.0) [44]), were used to analyse the stability of the expression of the internal RGs in different groups of samples. When using geNorm and NormFinder, the original Ct values were converted to 2^−ΔCt^ values (delta Ct = original Ct-value − the lowest Ct-value in each group) and used for stability analysis of the internal RGs. For BestKeeper, the E values and original Ct values were used to calculate the CV and SD of candidate internal RG expression. In geNorm, 2^−ΔCt^ values were also used as original data to determine the appropriate internal RG numbers by calculating the paired difference value (V_n_/V_n+1_) of two consecutive normalisation factors. In addition, the geometric means ranked by delta Ct, geNorm, NormFinder and BestKeeper were assessed to comprehensively evaluate RG stability, and the RefFinder program (https://www.heartcure.com.au/reffinder/?type=reference (accessed on 25 June 2019)) was used to verify the comprehensive ranking results.

### 4.6. Validation of RGs by qRT-PCR

It has been reported that miR396, miR164 and miR168 play an important role in plant growth and development and in response to (a)biotic stresses [36,37,38,39,40,45]. Thus, three miRNAs (aly-miR164c-5p, aly-miR168a-5p and smo-miR396) were selected as target genes; the primers used are shown in Table 1. The most stable and unstable internal RGs (combinations) were applied as references, and the expression levels of these target genes were calculated using the 2^−ΔΔCt^ method [46] to evaluate the reliabilities of the selected candidate RGs.

## 5. Conclusions

In summary, this study for the first time systematically and comprehensively examined the selection and evaluation of 13 candidate RGs for miRNA expression normalisation under six abiotic/hormone stresses in *C. fortunei.* Although no RG was the best under all experimental conditions, novel16, cln-miR6725, novel1 and *U6* are proposed as good choices for *C. fortunei* miRNA expression studies. Other reference miRNAs can be selected as well, but which is the most suitable depends on experimental treatment. In short, the findings of this study can be used for accurate and far-reaching miRNA expression analyses in *C. fortunei* under different conditions, as well as lay a foundation for studying its miRNA function in response to (a)biotic stresses.

## Figures and Tables

**Table 1:** Gene sequence and primer information.

Gene Symbol	Gene Sequence	Primer Sequence (5′-3′)	Amplicon Length (bp)	E (%)	R^2^
pab-miR159a	UUGGUUUGAAGGGAGCUCUAC	CGTTGGTTTGAAGGGAGCTCTAC	80–150	103.87	0.991
cln-miR162	UUGAUAAACCUCUGCAUCCAG	CGTTGATAAACCTCTGCATCCAG		113.42	0.978
cas-miR166d	UCGGACCAGGCUUCAUUCCCCU	TCGGACCAGGCTTCATTCC		108.38	0.997
pab-miR395b	CAGAAGUGUUUGGGGGGACUC	CAGAAGTGTTTGGGGGGACTC		112.25	0.985
ppt-miR894	CGUUUCACGUCGGGUUCACC	GTTTCACGTCGGGTTCACC		103.55	0.979
cln-miR6725	UGGCAUCUGUCGAGGUCAUCUA	TGGCATCTGTCGAGGTCATCTA		110.67	0.982
novel1	UCUUUCCGGAUCCUCCCAUGCC	CTTTCCGGATCCTCCCATGC		104.29	0.999
novel6	UUUUACCGAUCCCUCCAAAGCC	TTTACCGATCCCTCCAAAGCC		92.93	0.993
novel14	UUUGAGUGAAUCCAGAGUCUCU	CGCTTTGAGTGAATCCAGAGTCTC		90.04	0.995
novel16	UUUUUCCAAUACCUCCUAUACC	GCGTTTTTCCAATACCTCCTATACC		92.93	0.993
*U6*	–	Forward: ACAGAGAAGATTAGCATGGCC	59	114.97	0.995
		Reverse: GACCAATTCTCGATTTGTGCG			
*5S*	–	Forward: CACCAATCCATCCCGAACTT	193	97.98	0.998
		Reverse: CCGTCTCCACCAGATAACAAATA			
*18S*	–	Forward: TCTGGTCCTGTTCCGTTGG	124	100.36	0.995
		Reverse: GCTTTCGCAGTGGTTCGTC			
Target genes					
aly-miR164c-5p	UGGAGAAGCAGGGCACGUGCG	TGGAGAAGCAGGGCACG	80–150	110.09	0.994
aly-miR168a-5p	UCGCUUGGUGCAGGUCGGGAA	TCGCTTGGTGCAGGTCG		115.20	0.987
smo-miR396	UUCCACGGCUUUCUUGAACC	GTTCCACGGCTTTCTTGAACC		104.24	0.997

**Table 2:** Expression-stability values and the ranking of reference genes calculated by NormFinder.

Treatment		1	2	3	4	5	6	7	8	9	10	11	12	13
Cold	Gene	novel16	novel1	miR6725	miR159a	novel14	*U6*	miR395b	*5S*	miR894	novel6	*18S*	miR162	miR166d
	Stab.	0.187	0.210	0.213	0.318	0.360	0.386	0.404	0.448	0.491	0.605	0.655	0.805	0.859
Heat	Gene	novel16	miR159a	*U6*	novel1	miR166d	novel6	*5S*	miR395b	miR6725	miR162	miR894	novel14	*18S*
	Stab.	0.349	0.364	0.388	0.428	0.452	0.509	0.540	0.606	0.620	0.699	0.881	0.881	0.941
Drought	Gene	novel16	*U6*	novel1	miR159a	novel6	miR6725	novel14	*18S*	miR395b	miR162	miR894	*5S*	miR166d
	Stab.	0.155	0.254	0.255	0.338	0.425	0.430	0.432	0.503	0.646	0.806	0.912	1.041	1.181
Salt	Gene	novel16	miR6725	miR395b	novel1	miR159a	*U6*	*5S*	novel6	*18S*	novel14	miR894	miR166d	miR162
	Stab.	0.219	0.267	0.347	0.359	0.386	0.434	0.439	0.476	0.534	0.587	0.832	0.913	1.027
ABA	Gene	*U6*	novel16	novel1	miR159a	miR6725	miR894	novel6	miR395b	*5S*	novel14	*18S*	miR166d	miR162
	Stab.	0.193	0.211	0.220	0.239	0.243	0.283	0.306	0.328	0.365	0.370	0.587	0.684	0.917
GA_3_	Gene	miR6725	novel1	*18S*	miR159a	novel16	*5S*	novel6	*U6*	novel14	miR395b	miR894	miR162	miR166d
	Stab.	0.176	0.271	0.310	0.312	0.366	0.452	0.461	0.560	0.570	0.684	0.782	0.790	1.314
Treatment	Gene	novel16	miR159a	miR6725	novel1	*U6*	novel6	miR395b	*18S*	novel14	miR162	*5S*	miR894	miR166d
	Stab.	0.280	0.344	0.356	0.372	0.373	0.410	0.563	0.586	0.588	0.733	0.737	0.816	0.864
Tissue	Gene	novel1	novel6	novel16	miR6725	novel14	*18S*	miR395b	*U6*	miR162	miR166d	*5S*	miR894	miR159a
	Stab.	0.279	0.336	0.404	0.511	0.523	0.607	0.677	0.756	0.889	1.060	1.123	1.178	1.344
Total	Gene	novel16	miR6725	novel1	novel6	*U6*	miR159a	miR395b	*18S*	novel14	miR162	*5S*	miR894	miR166d
	Stab.	0.304	0.373	0.381	0.390	0.452	0.556	0.601	0.612	0.634	0.789	0.799	0.876	0.877

Stab., stability value; miR159a, pab-miR159a; miR162, cln-miR162; miR166d, cas-miR166d; miR395b, pab-miR395b; miR894, ppt-miR894; miR6725, cln-miR6725.

**Table 3:** Top five genes ranked by various algorithms.

Software	Rank	Cold	Heat	Drought	Salt	ABA	GA_3_	Treatment	Tissue	Total
delta Ct	1	cln-miR6725	novel1	novel16	novel16	*U6*	cln-miR6725	novel16	novel1	novel16
	2	novel16	novel16	*U6*	cln-miR6725	novel16	novel1	pab-miR159a	novel6	novel6
	3	novel1	pab-miR159a	novel1	pab-miR395b	novel1	*18S*	cln-miR6725	novel16	novel1
	4	pab-miR159a	cas-miR166d	pab-miR159a	novel1	cln-miR6725	pab-miR159a	novel1	cln-miR6725	cln-miR6725
	5	pab-miR395b	novel6	novel6	*5S*	pab-miR159a	novel16	*U6*	novel14	*U6*
geNorm	1	novel16	novel1	novel6	*5S*	novel16	novel1	novel16	novel16	novel16
	2	cln-miR6725	pab-miR395b	pab-miR159a	*U6*	novel1	cln-miR6725	pab-miR159a	cln-miR6725	cln-miR6725
	3	novel1	*5S*	*U6*	*18S*	cln-miR6725	*18S*	cln-miR6725	novel1	novel1
	4	pab-miR159a	novel6	novel16	pab-miR395b	*U6*	pab-miR159a	novel1	novel6	novel6
	5	novel14	cas-miR166d	novel1	novel6	pab-miR159a	novel6	*U6*	*18S*	*U6*
NormFinder	1	novel16	novel16	novel16	novel16	*U6*	cln-miR6725	novel16	novel1	novel16
	2	novel1	pab-miR159a	*U6*	cln-miR6725	novel16	novel1	pab-miR159a	novel6	cln-miR6725
	3	cln-miR6725	*U6*	novel1	pab-miR395b	novel1	*18S*	cln-miR6725	novel16	novel1
	4	pab-miR159a	novel1	pab-miR159a	novel1	pab-miR159a	pab-miR159a	novel1	cln-miR6725	novel6
	5	novel14	cas-miR166d	novel6	pab-miR159a	cln-miR6725	novel16	*U6*	novel14	*U6*
BestKeeper	1	novel14	novel1	*U6*	novel16	*U6*	*U6*	*U6*	novel1	*U6*
	2	cln-miR6725	*U6*	novel16	cln-miR6725	ppt-miR894	cln-miR6725	pab-miR159a	*U6*	novel1
	3	novel16	novel16	cln-miR6725	novel1	novel1	*5S*	novel16	*18S*	novel16
	4	pab-miR159a	cas-miR166d	novel14	pab-miR395b	*5S*	novel14	novel1	cln-miR6725	cln-miR6725
	5	pab-miR395b	pab-miR395b	pab-miR159a	pab-miR159a	cln-miR6725	novel16	cln-miR6725	novel16	pab-miR159a
RefFinder	1	cln-miR6725	novel1	novel16	novel16	*U6*	cln-miR6725	novel16	novel1	novel16
	2	novel16	novel16	*U6*	cln-miR6725	novel16	novel1	pab-miR159a	novel16	cln-miR6725
	3	novel1	*U6*	pab-miR159a	pab-miR395b	novel1	*18S*	*U6*	cln-miR6725	novel1
	4	novel14	pab-miR159a	novel1	*5S*	cln-miR6725	*U6*	cln-miR6725	novel6	*U6*
	5	pab-miR159a	pab-miR395b	novel6	*U6*	ppt-miR894	*5S*	novel1	*18S*	novel6

## Supplementary Materials

The following are available online at https://www.mdpi.com/article/10.3390/ijms22147246/s1, Table S1: transcripts per million (TPM) values in *C. fortunei* needles.
