# Supplementary material for: Selection and Optimization of Reference Genes for MicroRNA Expression Normalization by qRT-PCR in Chinese Cedar (Cryptomeria fortunei) under Multiple Stresses
Source: Int J Mol Sci. 2021 Jul 6;22(14):7246. doi: 10.3390/ijms22147246 (PMC8304282; doi:10.3390/ijms22147246)
Supplement: Supplementary file 1 [file ijms-22-07246-s001.zip › ijms-1289537-supplementary.pdf]

Table S1. Transcripts per million (TPM) values in *C. fortunei* needles.

miRNA_id	YWt1	YWt2	YWt3
pab-miR159a	46.70	80.97	84.71
cln-miR162	1134.10	985.82	1057.89
cas-miR166d	1146.86	2144.01	1743.46
pab-miR395b	435.66	491.13	540.71
ppt-miR894	19.869	24.28764716	19.89
cln-miR6725	41.60	38.83788794	28.78
novel1	911.93	2159.622361	1952.78
novel6	936.42	1010.462585	646.74
novel4	795.63	673.8579285	615.08
novel16	203.33	248.7506057	150.02

YWt1, YWt2 and YWt3 represent #3 needles in November, December and February, respectively. TPM values were calculated to represent miRNA expression levels, and values in the table are the average of 3 replicates (n = 3) values.
